# The Clinical Characteristics and Surgical Outcomes of Epiblepharon in Korean Children: A 9-Year Experience

**DOI:** 10.1155/2014/156501

**Published:** 2014-09-14

**Authors:** Jong Soo Kim, Sang Wook Jin, Mun Chong Hur, Yoon Hyung Kwon, Won Yeol Ryu, Woo Jin Jeong, Hee Bae Ahn

**Affiliations:** Department of Ophthalmology, Dong-A University College of Medicine, 3ga, Dongdaeshin-dong, Seo-gu, Busan 602-715, Republic of Korea

## Abstract

*Purpose*. To examine the demographic characteristics, clinical features, surgical outcomes, and long-term prognoses of epiblepharon in Korean children. *Methods*. Epiblepharon patients who were followed for ≥ 6 month following surgical correction between January 2005 and December 2013. The patient demographics, clinical features, concomitant disorders, surgical outcomes, and complications were retrospectively reviewed. *Results*. A total of 768 epiblepharon records were included in the analysis. The mean patient age was 6.55 ± 2.37 years. At presentation, 712 patients (92.8%) complained of typical epiblepharon symptoms. The mean patient age at surgery was 6.95 ± 2.52 years, with 629 patients (81.9%) on the lower lid and 72 patients (9.4%) on the upper lid and 82 patients (10.7%) undergoing surgery on both lids. The eyelid was well everted with no recurrence in 740 patients (96.4%). *Conclusion*. Epiblepharon frequently occurs in Korean children and is correctable with a simple surgery. Recurrence and serious complications do not occur often, and any suspicions of epiblepharon should be investigated. A thorough ocular examination can lead to a correct diagnosis and timely corrective surgery. Most procedures are successful and prevent secondary complications that often occur with uncorrected epiblepharon.

## 1. Introduction

Epiblepharon is a relatively common eyelid anomaly frequently found in Asian infants and children. It is characterized by a horizontal skin fold across the edge of the eyelid, occurring more often in the lower lid [1–5]. The two known causes of lower eyelid epiblepharon are inadequate lower lid retractor development, marked by the absence of adhesion to the skin, and a pretarsal orbicularis muscle inserted too closely to the lid margin; subsequently, the muscle and skin anterior to the tarsal plate are pushed forward over the tarsal plate, resulting in muscle and skin hypertrophy. In contrast, the causes of congenital entropion, which differs from epiblepharon, are a lack of a tarsus, hypertrophy of the pretarsal orbicularis muscle in the eyelid, and an aponeurosis retractor attachment rupture [6, 7].

With redundant lower eyelid skin and insufficient adhesion of the orbicularis oculi muscle to the tarsal plate, the result is a displaced skin crease above the tarsal plate. This skin causes the cilia to be inverted toward the eyeballs, which may touch and irritate the cornea, particularly in the downward gaze [1, 2]. Associated symptoms of this condition include ocular irritation (e.g., epiphora, photophobia, eye rubbing, increased discharge, frequent blinking, and foreign body sensation) and corneal erosion, both of which can result in vision impairment [8, 9].

In most cases, symptoms are not serious and are outgrown with facial bone growth and the accompanying skin and muscle extension. However, in serious cases or when the condition persists, surgical correction is often necessary [10, 11]. Compared to Caucasians, Asians generally have nasal bones that tend to rise at a later age, and it has been reported that 12.6% of Asian children aged 7 to 14 years old have epiblepharon. The condition does not always correct itself with age, and surgical correction should be performed once symptoms appear [6].

Despite numerous studies examining the prevalence of epiblepharon in Asian populations [12–14], no large-scale, long-term studies on epiblepharon in Korean infants and children have been previously performed. Here, we report on the prevalence, symptoms and surgical success, recurrence, and complication rates observed in Korean children undergoing epiblepharon surgical correction. Patients were followed, on average, for just over 1 year following surgery.

## 2. Materials and Methods

Among our patients, 768 patients were diagnosed with epiblepharon, underwent corrective surgery between January 2005 and December 2013, and were followed for at least 6 months following surgery.

Patient medical records were retrospectively reviewed for the age, gender, chief complaint, presence of keratitis, location of epiblepharon, concomitant disorders, postoperative location of epiblepharon, complications, and recurrence rate. The success rate of repeat procedures was also examined, where applicable. Epiblepharon was classified by the location into the upper lid and the lower lid groups. Additionally lower cilia-touching lesions were classified into nasal, center, and temporal groups, according to the corneal region involved.

Surgery was performed when significant corneal damage occurred, as confirmed by slit lamp examination or serious irritation complaints. The surgeries were performed by the same surgeon and involved skin resection and full-thickness rotating suturing in the lower lid corrections and simple-interrupted buried suturing in the upper lid corrections. Skin resection and full-thickness rotating suture placement on the lower lid involved marking a skin incision line with the aid of Bishop forceps. After an incision was made with a number 15 Bard-Parker blade, skin and subcutaneous tissue resection was performed with Stevens scissors. Part of the orbicularis oculi was also removed using electrocautery to minimize bleeding. After exposing the tarsal plate, one or two simple-interrupted buried sutures (6-0 nylon) were placed in the subcutaneous tissues of the incision's upper end and on the lower end of the tarsal plate, with the resected skin inserted into the incision. For the remaining skin, a continuous suture (6-0 fast absorbing plain gut) was placed.

The simple-interrupted buried suture was also placed for the upper lid correction, paying close attention to bilateral lid symmetry. Four points were marked with a marking pen where the double fold was to be formed and the stitches were to be placed (Figure 2). Epinephrine mixed with 2% lidocaine was injected into the subcutaneous space and the upper subconjunctival layers. Several 2-3* *mm incisions were then made with a number 11 blade for the suture entry. The upper lid was everted and a single-interrupted buried suture (4-0 vicryl) was placed on the 4 marked points.

All postoperative follow-up exams were performed in an outpatient setting in which the position of the cilia and cilia-corneal touch were monitored by slit lamp examinations. During the follow-up period, the patients were carefully monitored for surgical complications and epiblepharon recurrence.

## 3. Results

**Figure 1:**
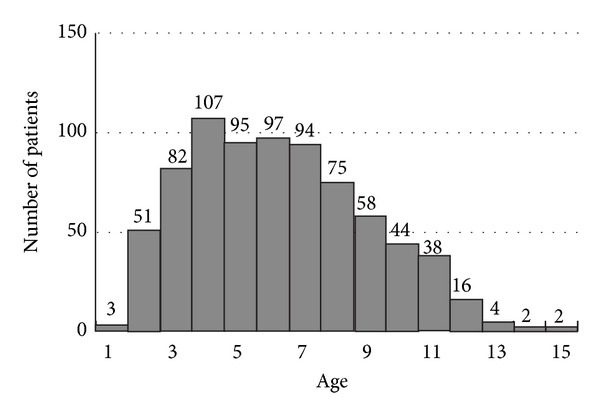
Distribution of patients.

**Figure 2:**
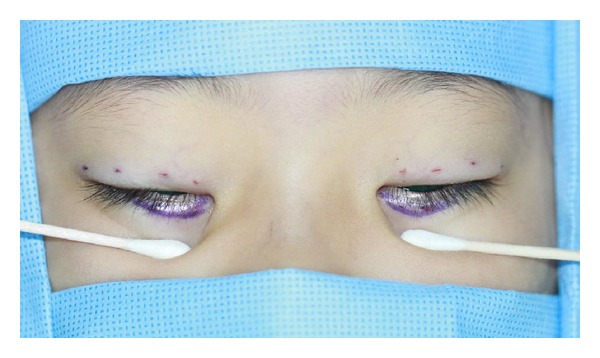
The photography showing the marking of the upper lid sutures placement and the lower lid incision.

Among the 768 epiblepharon patients included, 302 (39.3%) were male and 466 (60.7%) were female. The mean patient age at the time of presentation was 6.55 ± 2.37 years (Table 1). Four-year-olds composed the largest patient group (*n* = 107, 13.9%), with the majority of patients aged between 3 and 7 years (*n* = 475, 61.8%, Figure 1 and Table 2). At the time of presentation, 712 patients (92.8%) reported epiblepharon symptoms, but 56 patients (7.3%) were incidentally discovered while undergoing visual acuity and/or ophthalmologic examinations for other reasons. The most common complaints among patients were foreign body sensation due to cilia-corneal touch (*n* = 209, 27.2%), ocular discharge (*n* = 97, 12.6%), photophobia (*n* = 80, 10.4%), and decreased visual acuity (*n* = 69, 9.0%, Table 3). Lower lid epiblepharon patients had cornea-cilia touch most often in the nasal area (*n* = 408, 58.6%), followed by the central area (*n* = 264, 37.9%), and, finally, the temporal area (*n* = 24, 3.4%, Table 1). The concomitant eye disorders were ptosis (*n* = 62, 8.1%), strabismus (*n* = 48, 6.3%), floppy eyelid syndrome (*n* = 44, 5.8%), trichiasis (*n* = 37, 4.8%), and amblyopia (*n* = 10, 1.3%, Table 4).

The mean patient age at the time of surgery was 6.95 ± 2.52 years. Epiblepharon correction surgery was performed on both the upper and lower lids of 82 patients (10.5%), on the lower lids of 629 patients (80.3%), and on the upper lids of 72 patients (9.2%, Table 1). The mean postoperative follow-up period was 13.40 ± 7.58 months. Eyelid shape and function were maintained in 740 patients (96.4%) with no epiblepharon recurrence at the final follow-up examination.

The remaining 28 patients (3.6%) had epiblepharon recurrence involving the lower lid, 15 (1.9%) of whom were operated on a second time. Following the second procedure, no complications (e.g., ectropion, scar formation, and wound dehiscence) were observed (Table 5). The 13 patients who did not undergo a second correction procedure showed improvement without surgical intervention through the follow-up period. Statistically, the recurrence rate of lower cilia-corneal touch was significantly higher in the nasal area compared to the other corneal regions (Table 5).

## 4. Discussion

Epiblepharon is an eyelid anomaly common to Asian populations. It is generally a bilateral anomaly of the lower lid and is characterized by a skin fold that runs horizontally across the eyelid edge [1, 2, 15]. In their study of Japanese children, Noda et al. [15] reported that 81% of epiblepharon cases involved the lower lid and that only 7% and 12% of cases involved the upper lid or both lids, respectively. Fortunately, most infants outgrow epiblepharon, and symptoms spontaneously improve as facial bones, skin, and muscles grow. Only approximately 2% of infants with epiblepharon show no symptomatic improvement by the time they reach school age [15–17]. Because epiblepharon pushes the cilia toward the cornea and/or conjunctiva, symptoms include conjunctival irritation and injury, eye rubbing, and epiphora. It has also been associated with astigmatism and decreased visual acuity [9, 18].

In this study, the most common symptom at the time of presentation was cilia-induced corneal irritation, even in patients in whom severe epiblepharon was incidentally discovered (7.3%). Epiblepharon was observed most frequently in patients aged 4 to 7 years, which was indicative of the high prevalence rate for this age group. Therefore, even in infants without noticeable symptoms, careful examination is necessary to detect epiblepharon and prevent subsequent refractive error and decreased visual acuity caused by corneal irritation.

Epiblepharon that is mild or in an early stage may be conservatively addressed either with medication [19] or by retracting the skin fold downward with adhesive or cellophane tape [16, 20]. Surgical intervention criteria vary slightly between surgeons, but, in most cases, the presence of symptomatic cilia-induced corneal irritation is the decisive factor [12, 21]. However, some controversy remains regarding this issue because even in the presence of corneal irritation, some infants and children do not complain, and more conservative therapies can be used successfully. In the current study, the severity of corneal irritation was subjectively assessed by a single surgeon to determine whether surgical intervention was needed. No objective criteria for this evaluation have been established, although such criteria would have been of great use and an interesting area for further study.

Epiblepharon correction methods are broadly divided into nonincisional and incisional methods [22–24]. Nonincisional methods result in less postoperative edema, can easily be repeated, and leave minimal scars; unfortunately, these methods result in occasional recurrence [25, 26]. In this study, the nonincisional simple-interrupted buried suture technique was used to correct upper lid epiblepharon, and no recurrence was observed during the follow-up.

Lower lid epiblepharon correction methods include several slightly varied techniques. The nonincisional suture technique, first presented by Quickert et al. [6], is simple and rapid with a reported success rate of 100%. Nevertheless, the disadvantages of this technique make it impractical for severe epiblepharon correction and include frequent suture infection and a recurrence rate of up to 29%. The modified Hotz procedure (i.e., Hotz-Celsus procedure) was widely used until recently. It involves lower lid redundant skin and partial orbicularis oculi resection and placing an anchoring suture of skin to the tarsal plate. It is a relatively simple procedure with a high success rate but requires an unnecessarily large skin resection, which can result in ectropion and lid retraction [4, 25, 26]. The rotating suture technique can also be successful and generally has low complication rates. This procedure involves exposing the tarsal plate and burying it together with subcutaneous tissues [21, 27]. Our surgical method for lower lid epiblepharon correction involved only little skin resection, and when further correction was needed, the full-thickness rotating suture technique was used. Thus, lower lid crease, scar formation, and complication rates (e.g., ectropion and lid retraction) could be reduced, all while maximizing surgical success rate and minimizing recurrence rate.

Our recurrence and reoperation rates were highest in patients with lower lid epiblepharon with nasal cilia-cornea touch. This result was in agreement with Swan [28], who found that, in severe epiblepharon, creasing at the nasal end of the eyelid occurs substantially more frequently. Kwon et al. [29] reported on an additional epiblepharon surgical technique that, coupled with Z-epicanthoplasty, positively affected defect correction. In our study, in an attempt to overcome the defect, an additional full-thickness rotating suture was placed in cases of severe epiblepharon. Still, recurrence could not be prevented in all cases. Therefore, further research is necessary to identify the most effective intervention method that reduces recurrence and reoperation rates by sufficiently improving cases of severe epiblepharon at high risk of recurrence.

The greatest limitation of this study was the subjective assessment for the need of surgery, but the influence of this limitation was minimized by having only one evaluating surgeon. Unfortunately, no objective system to evaluate cilia-induced corneal irritation symptoms and epiblepharon severity is available, and this system is a needed area of future research.

## 5. Conclusion

To conclude, epiblepharon is frequently found in Korean infants and children and should be addressed with caution and care, even in the absence of symptoms. The adverse effects of severe corneal irritation symptoms should also be considered. Many surgical interventions carry the risks of general anesthesia and of postoperative complications, such as postoperative scar formation and ectropion. Here, we reinforce the idea that effective epiblepharon correction can be achieved with a simple surgery, alleviating symptoms and preventing keratitis-induced decreases in visual acuity. Nevertheless, age and symptom severity should be considered when determining which surgical correction method to use.

## Figures and Tables

**Table 1:** Patient demographic characteristics.

Characteristic	Description
M : F (*n*)	302 (39.3%) : 466 (60.7%)
Mean age (yrs)	6.55 ± 2.37
Location of epiblepharon	
Upper + lower	82 (10.5%)
Upper	72 (9.2%)
Lower	629 (80.3%)
Lower cilia touched lesion	
Nasal	408 (58.6%)
Center	264 (37.9%)
Temporal	24 (3.4%)
Mean age at operation (yrs)	6.95 ± 2.52
Mean follow-up time (months)	13.40 ± 7.58

**Table 2:** Chief complaint of patients.

Chief complaint	Patient (%)
Foreign body sensation due to cilia touched cornea	209 (27.2%)
Discharge	97 (12.6%)
Photophobia	80 (10.4%)
Decreased visual acuity	69 (9.0%)
Eye rubbing	68 (8.9%)
Epiphora	57 (7.4%)
Incidental	56 (7.3%)
Conjunctival injection	47 (6.1%)
Itching	44 (5.7%)
Frequent blinking	41 (5.3%)

**Table 3:** Associated ocular disease in epiblepharon patients.

Associated anomaly	Patient (%)
Ptosis	62 (8.1%)
Strabismus	48 (6.3%)
Floppy eyelid syndrome	44 (5.8%)
Trichiasis	37 (4.8%)
Amblyopia	10 (1.3%)

**Table 4:** Postoperative results.

	Patient (%)
Well corrected	740 (96.4%)
Recurrence	28 (3.6%)
Reoperation	15 (1.9%)
Improvement of symptoms without reoperation	13 (1.7%)
Postoperative complication (e.g., ectropion and lid retraction)	0 (0%)

**Table 5:** Distribution of surgical outcome according to lower cilia touched lesion.

Lower cilia touched lesion	Patient (%)			
	Success	Recurrence		Total
		Reoperation	Observation	
Nasal	384 (94.1%)	13 (3.2%)	11 (2.7%)	408
Center	260 (98.5%)	2 (0.8%)	2 (0.8%)	264
Temporal	24 (100%)	0 (0%)	0 (0%)	24
*P* value	0.003∗		0.028^†^	

*P value of Pearson's chi-squared test (linear-by-linear association) between recurrence and lower cilia touched lesions.

^†^ *P* value of Pearson's chi-squared test (linear-by-linear association) between reoperation and lower cilia touched lesions.
